# Integrated Multi-Omics Reveals Gut Microbiota-Ovarian Crosstalk of Laying Performance Between High- and Low-Laying Muscovy Ducks

**DOI:** 10.3390/vetsci13080816

**Published:** 2026-08-17

**Authors:** Xiujun Duan, Rui Zhu, Youqing Bian, Xiaoming Li, Guobo Sun, Yue Wang, Qingyi Zhou, Lei Zhang

**Affiliations:** 1 School of Modern Animal Husbandry, Jiangsu Agri-Animal Husbandry Vocational College, Taizhou 225300, China; 2 Jiangsu Key Laboratory for High-Tech Research and Development of Veterinary Biopharmaceuticals, Engineering Technology Research Center for Modern Animal Science and Novel Veterinary Pharmaceutic Development, Taizhou 225300, China

**Keywords:** cecal microbiota, Muscovy duck, egg production, follicle development, *Ligilactobacillus*

## Abstract

As a high-value meat duck breed, Muscovy ducks exhibit marked individual differences in egg production that limit the economic efficiency of commercial farming. To elucidate the underlying mechanisms, we integrated phenotypic observation, ovarian transcriptome sequencing, and cecal 16S rRNA gene amplicon sequencing to compare high- and low-laying black-feathered Muscovy duck populations. We recorded 40-day egg production, assessed ovarian morphology, quantified gene expression profiles, and characterized microbial community structure. High-laying ducks produced significantly more eggs (24.60 ± 9.22) than low-laying ducks (15.80 ± 6.64, *p* < 0.01), with larger ovaries and a greater number of follicles at all developmental stages. Transcriptomic analysis revealed 823 DEGs, with KEGG enrichment implicating pathways governing ovarian physiology, including MAPK, Calcium, Notch, and Wnt signaling. Microbial profiling demonstrated significant differences in α/β diversity between groups, with high-laying ducks exhibiting an elevated *Firmicutes*/*Bacteroidota* ratio and decreased abundance of *Ligilactobacillus*. Integrative correlation analysis identified significant associations between *Ligilactobacillus* abundance and the expression of *Arhgap27* and *HOXA10*, two genes linked to reproductive function, and highlighted the glycolysis/gluconeogenesis pathway as a convergently enriched signaling cascade shared between gut microbiome and ovarian transcriptome. This multi-omics study uncovers the molecular and microbial basis of egg production variation in Muscovy ducks, offering potential targets for improving breeding efficiency through microbiota-directed or gene-based interventions. These findings deepen our understanding of host–microbe interactions in poultry reproduction and identify candidate genes and microbial taxa that may serve as potential targets for future nutritional or genetic interventions to enhance laying performance in commercial duck production.

## Simple Summary

Muscovy ducks are valuable meat-type poultry, but individual egg production varies greatly, impacting farm profitability. To explore the underlying causes, we compared high- and low-laying ducks using transcriptomic and gut microbial analyses. We identified 823 differentially expressed genes and distinct gut bacterial features between the two groups. High-laying ducks exhibited a higher *Firmicutes*/*Bacteroidota* ratio and lower *Ligilactobacillus* abundance, both correlating with ovarian gene expression. Integrative analysis further revealed that *Ligilactobacillus* abundance correlated with the reproduction-related genes *Arhgap27* and *HOXA10*, suggesting a potential gut–ovary molecular interface. These findings may inform breeding strategies to improve duck production efficiency.

## 1. Introduction

The Muscovy duck (*Cairina moschata*) cultivated in China traces its origins to the tropical zones of Central and South America [1]. This breed is highly valued in the poultry industry for its superior muscle quality, reduced subcutaneous fat deposition, increased proportion of breast and leg meat, and high crude protein content [2,3]. Nevertheless, substantial variations in reproductive output are observed across different flocks, attributable to differences in acclimatization to local environments, historical translocation routes, and genetic diversity. Under standard commercial management, the annual egg production of Chinese Muscovy ducks typically falls between 67 and 110 eggs per duck [4], a figure that remains far below the expectations for efficient mass rearing. In parallel, the species is characterized by an intense incubation instinct [5], which further compromises overall fecundity and poses a major bottleneck for the advancement of intensive operations. Given these constraints, deciphering the genetic architecture that underpins laying performance is of paramount importance, and leveraging this knowledge to develop genetic marker-assisted selection programs offers a promising avenue for genetic progress and industrial sustainability. As the primary reproductive organ governing egg formation, the ovary serves as the ideal tissue for probing the molecular determinants of laying performance of Muscovy ducks. In their transcriptomic work, Bao et al. [6] profiled ovarian gene expression patterns in high- versus low-producing individuals from both black and white Muscovy ducks. Their analyses unearthed a suite of differentially expressed genes (DEGs) (e.g., *TGFβ2*, *NGFR*, and *CEBPD*) putatively involved in ovarian ontogeny, with subsequent pathway enrichment pointing to the PI3K-Akt signaling cascade and ovarian steroidogenesis as the two most relevant regulatory networks governing reproductive efficiency. To complement the transcriptional insights at the protein level, Wu et al. [7] carried out a comparative proteomic analysis on ovarian tissues from laying versus broody Muscovy ducks. This investigation pinpointed six proteins (APOV1, GAL, SAA, GNB5, VLDLR, and CDK1) as candidates potentially relevant to reproductive performance, with the steroid biosynthesis pathway emerging as the most prominently enriched functional term, underscoring its putative role in ovarian function and reproductive status.

Beyond the ovary—long the classical focus of avian reproductive research—the gut microbiota has crystallized as a compelling frontier in understanding poultry fecundity. This paradigm shift was conceptually anchored by Qi et al. [8], who advanced the notion that the gut microbial community operates as a “functional endocrine organ,” modulating ovarian physiology via crosstalk with estrogens, androgens, and insulin. Subsequent empirical efforts have lent weight to this framework. Wu et al. [9] surveyed 101 chickens across 13 breeds using 16S rRNA amplicon sequencing, revealing that high-producing hens harbored enriched *Firmicutes*, *Proteobacteria*, and *Lactobacillus*, whereas low producers exhibited greater α-diversity and more complex communities—a pattern suggestive of divergent metabolic economies impinging on nutrient assimilation, hormonal balance, and egg output. Extending this inquiry to Muscovy ducks, Huang et al. [10] identified *Lactobacillus* as a core cecal probiotic whose abundance correlated negatively with ammonia and positively with laying rate. In a complementary comparison, Zhu et al. [11] documented that broody ducks displayed suppressed prolactin alongside elevated follicle-stimulating hormone (*p* < 0.05), with *ileal Rothia*, *Streptococcus*, and *Lactobacillus* negatively associated with broodiness, whereas *Turicibacter*, *Aliicoccus*, and *Facklamia* showed positive associations. Of note, the positive correlation between *Lactobacillus* and egg production has been consistently observed across studies [12], reinforcing its candidacy as a probiotic modulator of reproduction. Collectively, these findings indicate that gut microbiota influence poultry reproduction beyond nutritional and immune pathways, their metabolic output and signaling capacity, operating through the microbiota–gut–ovary axis, exert direct and indirect effects on ovarian function, opening new avenues for microbiota-targeted interventions in commercial production.

Therefore, the gut–ovary axis has emerged as a critical regulator of reproductive physiology in mammals and poultry; however, its role in Muscovy ducks remains unexplored. Herein, we tested the hypothesis that this axis similarly governs reproductive performance in this species. Based on long-term selective breeding outcomes from our research group, two phenotypically distinct groups of Black Muscovy ducks (*Cairina moschata*) were established: a high-reproduction group and an ordinary-reproduction group. We systematically compared ovarian morphology, ovarian transcriptomic profiles, and cecal microbial communities between the two groups. The primary objective of this study was to determine whether the microbiota–gut–ovary axis contributes functionally to reproductive regulation in ducks. Our findings are expected to provide a theoretical foundation for future strategies aimed at modulating gut microbiota and host gene expression to improve egg production traits in Muscovy ducks.

## 2. Materials and Methods

### 2.1. Experimental Design and Sample Collection

Based on previous selective breeding in our research group, two distinct populations of Black Muscovy ducks (*Cairina moschata*) have been established: a high-reproduction (a) group and an ordinary-reproduction (b) group. From each population, 30 healthy female ducks at 200 days of age were randomly selected for this experiment. All ducks were individually housed in separate cages at the National Gene Bank of Waterfowl Resources in Jiangsu, China, under standard management conditions. The housing environment maintained a natural photoperiod, with ad libitum access to feed and water throughout the experimental period. The experiment lasted 47 days in total, including a 7-day acclimation period followed by a 40-day formal trial period. At the conclusion of the trial, six ducks from each group were randomly selected for sample collection. Euthanasia was performed by carbon dioxide inhalation followed by exsanguination via cervical dislocation. After euthanasia, ovarian tissues and cecal digesta were carefully excised, immediately snap-frozen in liquid nitrogen, and stored at −80 °C for subsequent analyses. The composition and nutrient levels of the basal diet are provided in Supplementary Table S1.

### 2.2. Laying Performance

Under standardized husbandry conditions, eggs were collected manually at 16:00 on a daily basis over the entire 40-day trial. Laying records were maintained for each duck to determine the total number of eggs laid per individual during the experimental period. Upon completion of the trial, 60 valid production records were available for further statistical scrutiny.

### 2.3. Ovarian Follicles Analysis

Ovarian tissues from six representative individuals per group (12 in total) were collected and subjected to morphological analysis. Based on follicular diameter, ovarian follicles were categorized into five developmental stages: preovulatory follicles (F), large yellow follicles (LYF), small yellow follicles (SYF), large white follicles (LWF), and small white follicles (SWF), following the criteria established by Gilbert [13] and Zou et al. [14]. The number of F, LYF, SYF, LWF, and SWF was recorded for each individual. Between-group comparisons were subsequently conducted for each of these traits to identify potential differences between Group a and Group b.

### 2.4. Ovary Transcriptome Data Analysis

Three ovarian stroma samples were selected randomly from each group, and then the qualified total RNA was extracted and transported to Novogene Co., Ltd. (Beijing, China) for transcriptome sequencing (RNA-Seq). The libraries were sequenced on an Illumina HiSeq 2500 system sequencing platform (Illumina, Inc., San Diego, CA, USA). The clean reads were mapped to the Muscovy duck’s reference genome (GCA_048319975.1) using hisat2 (2.0.5) for the downstream analysis as previously described [15]. The differentially expressed genes (DEGs) between samples were identified using the DESeq2 R package (1.20.0) [16], and *p* < 0.05 and |foldchange| ≥ 1 were used as the criteria of significance of DEGs. GO term and KEGG pathway analyses were performed by clusterProfiler software (3.8.1) [17], and *p* < 0.05 was considered to be significantly enriched. The STRING database [18] was used to explore the interaction between DEGs. The sequencing raw data have been deposited into the Genome Sequence Archive in National Genomics Data Center (https://ngdc.cncb.ac.cn/) with accession number PRJCA044169.

### 2.5. Quantitative Reverse Transcription Polymerase Chain Reaction (qRT-PCR) Analysis

Four arbitrarily selected DEGs were analyzed by qRT-PCR to confirm the RNA-Seq data. Total RNA was extracted with TRIzol (Invitrogen, Carlsbad, CA, USA), and primer pairs were designed using Primer 5.0 (Supplementary Table S2). qPCR was performed on a CFX96 Real-Time System (Bio-RAD, Hercules, CA, USA). Each 20-μL qPCR reaction, containing 10 μL of 2 × ChamQ SYBR Master Mix (Vazyme, Nanjing, China), 0.4 μL of each primer, 2 μL of cDNA, and 7.2 μL of sterile water, was run on a Bio-Rad CFX96 system. The protocol included 95 °C for 30 s, followed by 40 cycles of 95 °C for 5 s and annealing for 30 s. Melting curves were generated to verify single-product specificity, and appropriate negative controls were incorporated in each plate. All samples were assayed in triplicate, and relative expression was determined by the 2^−ΔΔCt^ method [19] using *GAPDH* and *β-ACTIN* as reference genes.

### 2.6. Cecal Microbiota Analysis

Cecal genomic DNA was extracted using the QIAamp 96 PowerFecal QIAcube HT kit (QIAGEN, Hilden, Germany) following the supplied protocol, with DNA quality checked on 1% agarose gels. To obtain representative samples for sequencing, we pooled equal amounts of DNA from three individuals in each group, producing three pooled samples per treatment group. The V3–V4 region of the 16S rRNA gene was amplified with the primer set 341F/806R according to the PCR program described in [20]. Amplified products were separated on 2% agarose gels and purified using the QIAquick gel extraction kit (QIAGEN, Hilden, Germany). Amplicon sequencing was conducted on the Illumina NovaSeq 6000 platform (Illumina, San Diego, CA, USA), and the resultant data were processed by Novogene Co., Ltd. (Beijing, China). Sequencing reads have been deposited in the BIG Data Center’s Genome Sequence Archive with the accession number PRJCA044144.

Bioinformatic processing involved merging paired-end reads with FLASH (v1.2.11) to obtain raw tags, followed by stringent quality filtering with fastp (v0.23.1) to generate clean tags. Effective tags were produced by aligning clean tags against the Silva reference database. These effective tags were then clustered into ASVs at 95% sequence similarity using QIIME2 (v2022.02). Community functional profiles were predicted using Tax4Fun (v1.1.5). Associations between environmental factors and bacterial genera were assessed by Spearman’s correlation analysis implemented in the psych R package (v1.8.4) [21]. Alpha diversity indices were compared between the high- and ordinary-reproduction groups using both Student’s *t*-test and Wilcoxon rank-sum test. Beta diversity was quantified via Unweighted UniFrac [22] and Weighted UniFrac distance matrices [23], which were calculated based on species abundance profiles. Finally, Spearman’s rank correlation coefficients were calculated using the psych R package (v1.8.4) to assess the associations between environmental parameters and relative abundances of bacterial genera.

### 2.7. Statistical Analysis

For 16S sequencing data, the alpha and beta diversity analyses were evaluated by QIME2 (202202). The alpha and beta indices were compared across groups with Kruskal–Wallis and ANOSIM tests using R software (V3.4.3), respectively. All statistical analyses in this study were performed with Statistical testing using one-way analysis of variance (ANOVA). Results were expressed as mean ± standard deviation. * signify a significant difference between means (*p* < 0.05), ** signify an extremely significant difference between means (*p* < 0.01), and *** represents highly extremely significant correlation (*p* < 0.001).

### 2.8. Ethic Statement

The protocol was performed after the approval of the Committee on Experimental Animal Management of Jiangsu Agri-Animal Husbandry Vocational College, Taizhou, China (No. Jsahvc-2023-94), and every effort was made to minimize animal suffering during the experiments.

## 3. Results

### 3.1. Comparison of Individual Egg Production and Follicular Development Between Two Groups

**Figure 1:**
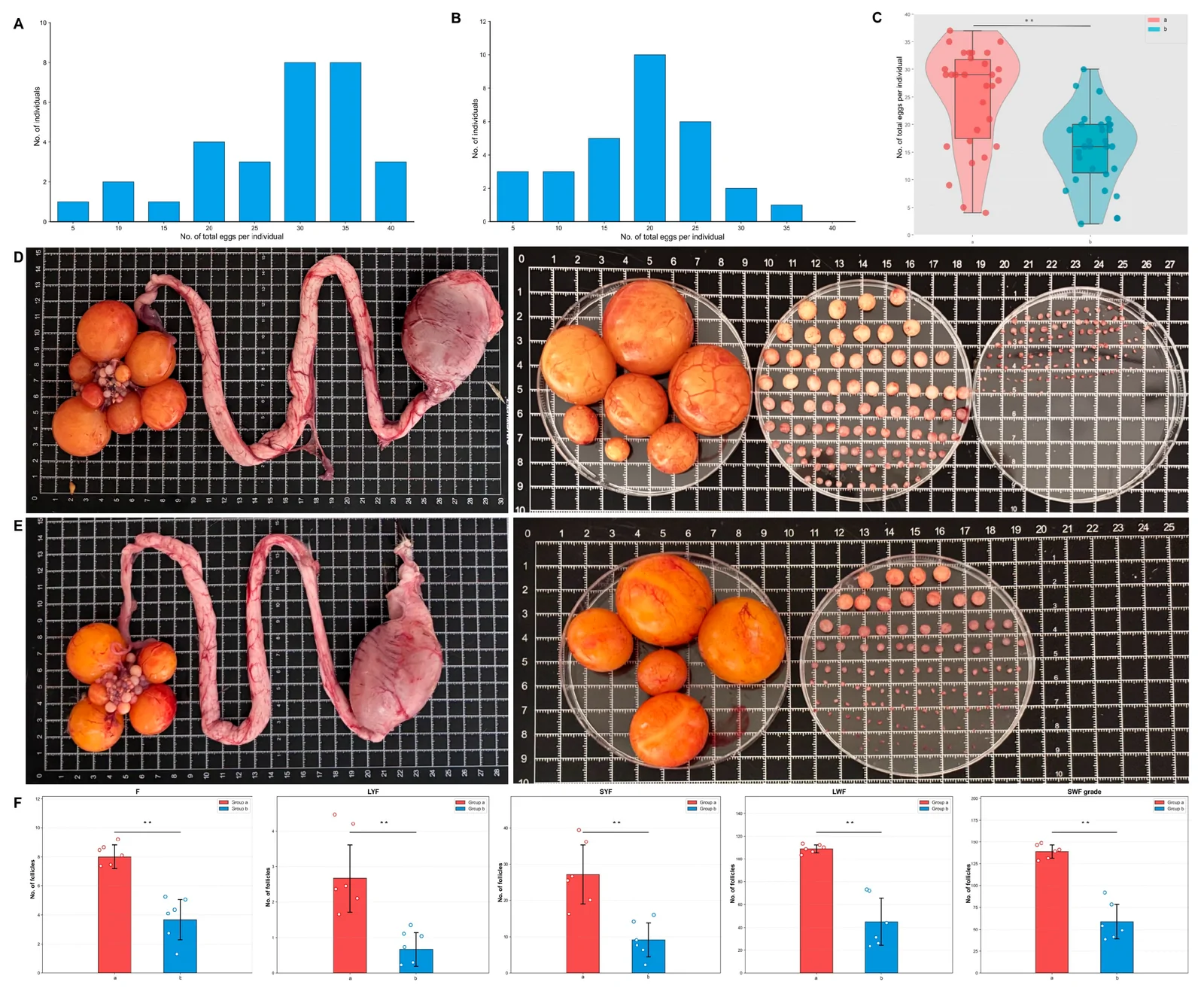
Egg production and follicle development characteristics in high− and low−laying groups. (**A**) Egg production in high− and low−laying groups. (**B**) Frequency distribution of egg production in the low−laying group. (**C**) Violin plot comparison of individual egg production between groups. (**D**) Ovarian morphology and graded follicles of the high−laying group. (**E**) Ovarian morphology and graded follicles of the low−laying group. (**F**) Statistical comparison of follicle numbers at different developmental stages. ** represents an extremely significant difference between means (*p* < 0.01).

To compare differences in caged egg production rates between the high-reproduction line and ordinary black-feathered Muscovy duck populations, we recorded the 40-day egg production quantity of each group. Based on the records, statistical analysis revealed that Muscovy ducks in Group a exhibited significantly higher individual egg production (24.60 ± 9.22 eggs) compared to those in Group b (15.87 ± 6.64 eggs, *p* < 0.01). Frequency distribution histograms indicated that egg production in Group A was concentrated primarily in the 20–30 egg range, while Group b values clustered between 10 and 20 eggs (Figure 1A,B), and violin plots further confirmed the significantly higher overall egg production and greater data variability in Group a (Figure 1C). Ovarian morphological observations showed larger ovarian volumes and more abundant multi-stage follicles in Group a (Figure 1D), in contrast to the smaller ovaries and fewer visible developing follicles in Group b (Figure 1E), with follicle grading statistics verifying that the quantity of follicles at all developmental stages was significantly higher in Group a (Figure 1F, *p* < 0.01).

### 3.2. Transcriptome Comparative Analysis and Functional Annotation

**Figure 2:**
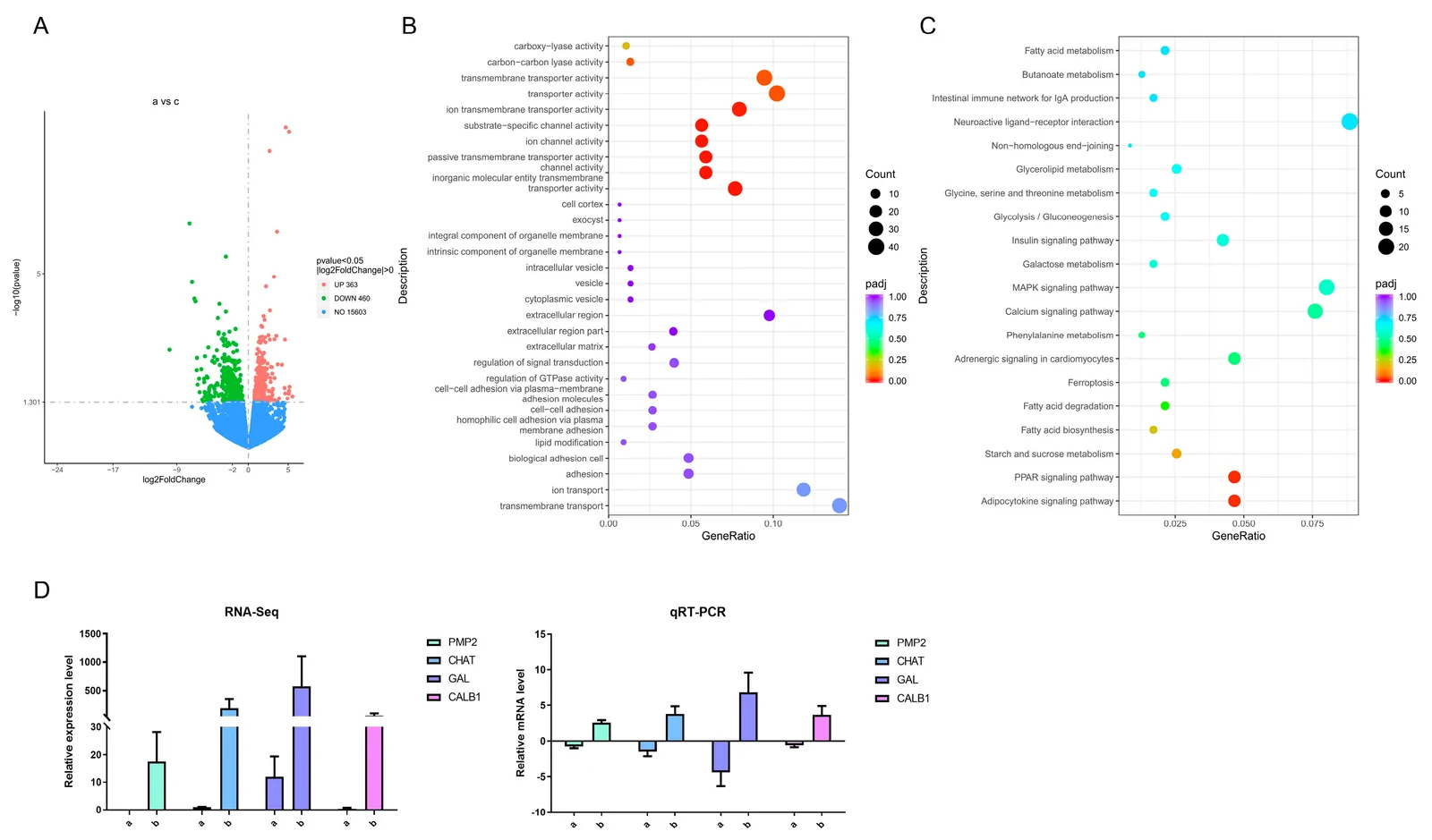
Identification and functional analysis of DEGs between high− and low−laying groups. (**A**) Volcano plot depicting DEGs identified by RNA−Seq; gray dots represent non−significant DEGs, while red and blue dots indicate significantly up− and down−regulated DEGs, respectively. (**B**) TOP 30 GO ontology classification based on DEGs between different dietary groups of caged laying ducks. (**C**) TOP 20 KEGG analysis based on DEGs between different dietary groups of caged laying ducks. (**D**) qRT−PCR validation plot for selected DEGs. The left panel shows RNA−Seq results, and the right panel shows qRT−PCR results for the same DEGs.

To investigate the molecular basis underlying differences in egg laying performance of Muscovy duck, the ovary transcriptomes between high- and low-laying groups were analyzed. As shown in Supplementary Table S3, 43,482,166~47,363,748 raw reads were obtained from these 6 samples, and correspondingly 41,633,694~44,230,232 clean reads were obtained from each sample after stringent quality control. The proportion of bases with an accuracy rate of >99.9% (Q30) in the total sequence after filtering exceeded 92.81%. Based on the positional information of gene alignments on the reference genome, the FPKM (Fragments per kilobase of exon model per million mapped fragments) of all genes in each sample was quantified. Collectively, these data attest to the robust quality and biological reproducibility, enabling their use in subsequent bio-informatics analyses. Genes with zero expression counts across all six samples were filtered out based on the database of known reference gene sequences and annotation files. Through comparative analysis, a total of 823 genes (363 upregulated and 460 downregulated) were differentially expressed between H and L groups, as shown in Figure 2A. GO enrichment analysis was performed to further express the functional roles of DEGs in different feed groups. The top 30 most significant GO terms in the pairwise comparisons are shown in Figure 2B. The most significant GO terms related to egg production in the a vs. b group included extracellular region, cytoplasmic vesicle, vesicle, and so on. The top 20 significantly enriched pathways by DEGs of each pairwise comparison are shown in Figure 2C, including fatty acid metabolism, glycerolipid metabolism, glycolysis/gluconeogenesis, and so on. qRT-PCR was applied to validate the transcriptional alterations of DEGs identified through RNA sequencing. Four DEGs were chosen at random for experimental detection. The expression tendencies of these genes measured by qRT-PCR matched well with the transcriptome results, proving the good credibility and precision of our sequencing data (Figure 2D).

### 3.3. Comparative Analysis and Functional Annotation of 16S rRNA Gene Amplicon Sequencing Data

**Figure 3:**
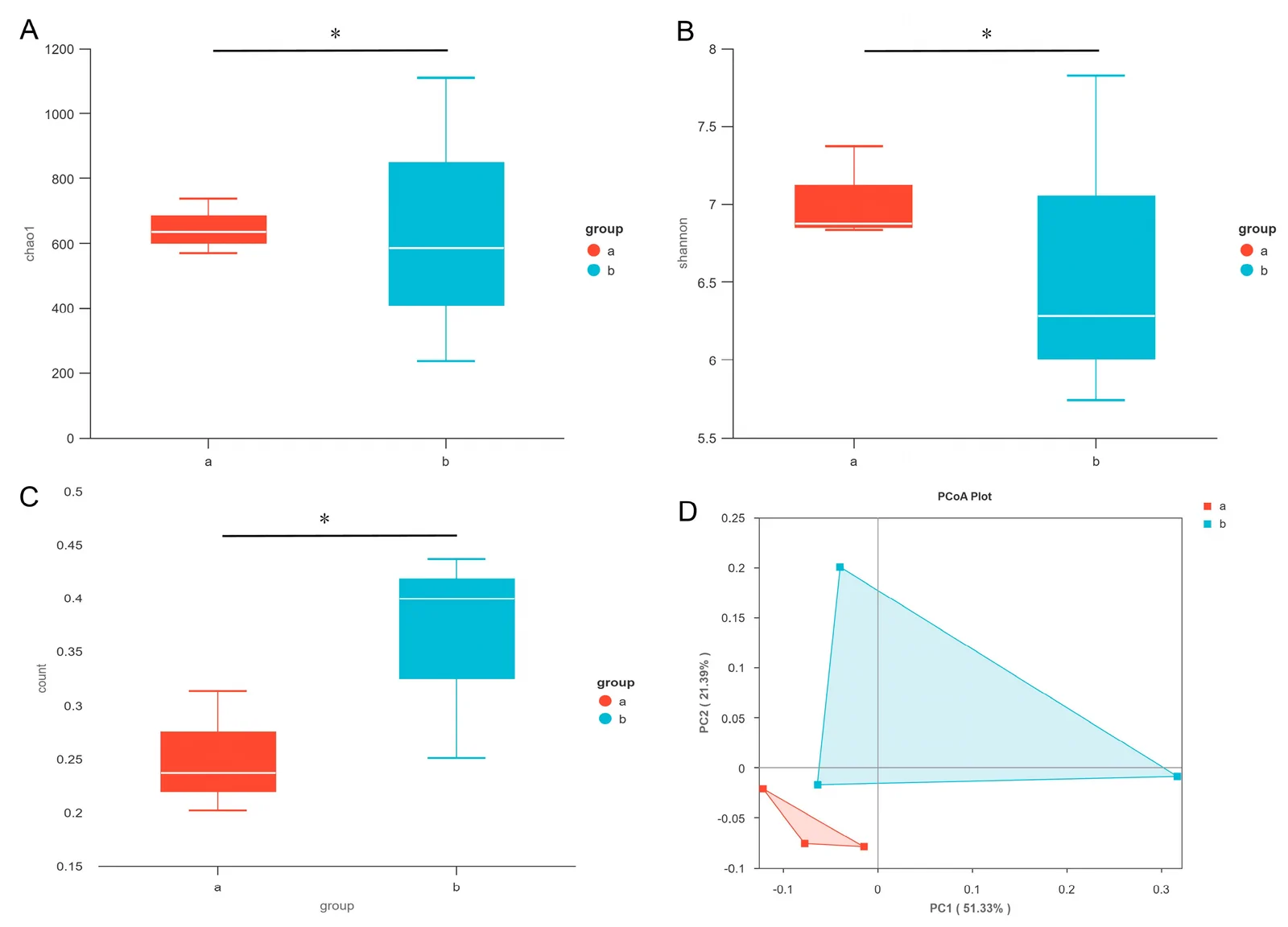
Alpha and beta diversity of cecal microbiota between high− and low−laying groups. (**A**) Chao1 index of cecal microbiota between two groups. (**B**) Shannon index of cecal microbiota between two groups. (**C**) Beta diversity index of cecal microbiota between two groups. (**D**) PCoA plot between two groups. * indicates statistical significance (*p* < 0.05).

After the initial filtering and adaptor trimming process, samples from high- and low-laying Muscovy duck groups accounted for a total of 546,108 qualified pair ends. After sequence assembly, data filtration and chimera removal, a total of 839,307 effective tags were obtained, which were classified into 448,039 effective tags (Nochime). The number of reads that passed through each step of assembly and control is presented in Supplementary Table S4. Based on species annotation results at different taxonomic levels (Phylum, Class, Order, Family, Genus, Species), bar charts were generated to display species annotation results corresponding to each sample at different taxonomic levels, as shown in Supplementary Figure S1. On this basis, alpha and beta diversity analyses of cecal bacterial communities between high- and low-laying Muscovy duck groups were performed respectively. A significant difference in the Chao1, Shannon, and beta diversity indices was observed between groups a and b (*p* < 0.05, Figure 3A–C). Principal Coordinates Analysis (PCoA) showed clear separation between groups a and b along the first principal component (PC1), which explained 51.33% of the total variance; PC2 explained an additional 21.39% of the variance. This distinct clustering pattern further confirmed significant differences in gut microbial community composition between the two groups (*p* < 0.05, Figure 3D).

**Figure 4:**
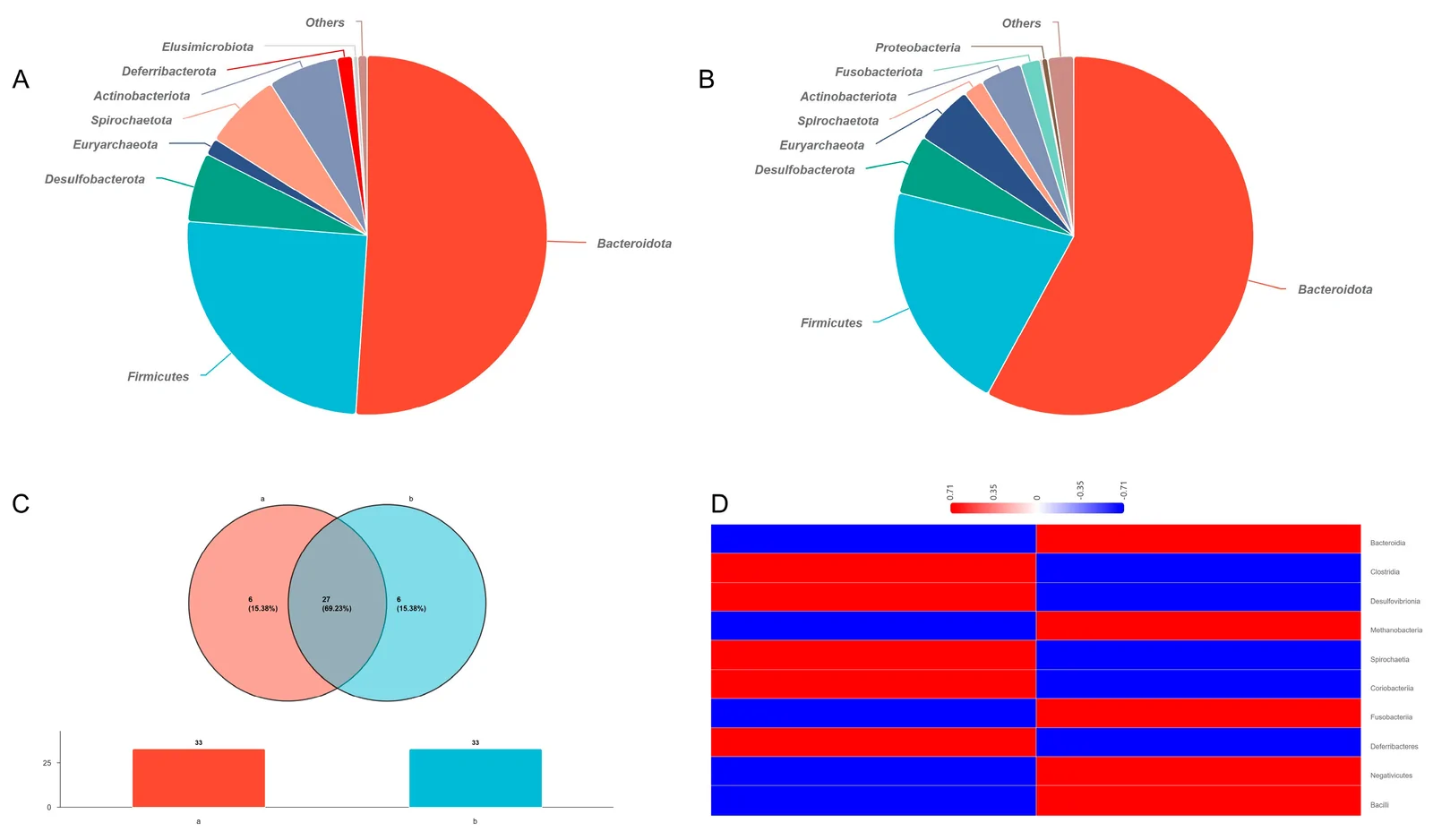
Relative abundance of the cecal microbiota at the phylum and class level between high− and low−laying groups. (**A**) Pie chart of the top 10 phyla with the highest relative abundance in Group a. (**B**) Pie chart of the top 10 phyla with the highest relative abundance in Group b. (**C**) Venn diagram analysis of shared and unique phyla between the two groups. (**D**) Heatmap of the top 10 classes with the highest relative abundance in the two groups.

**Figure 5:**
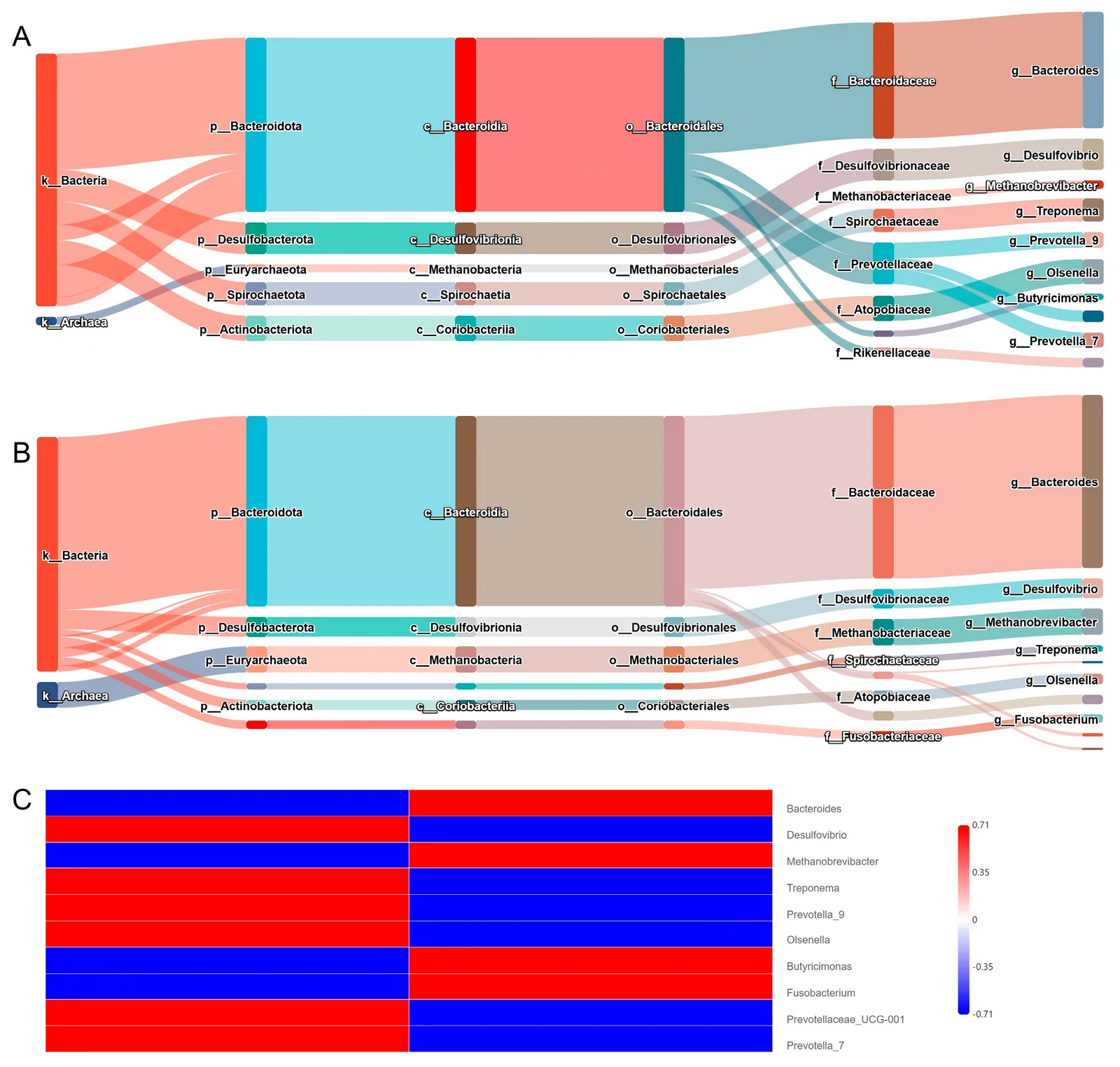
Relative abundance of the cecal microbiota at the genus level between high− and low−laying groups. (**A**) Sankey diagram of the top 10 genera with the highest relative abundance in Group a. (**B**) Sankey diagram of the top 10 genera with the highest relative abundance in Group b. (**C**) Heatmap of the top 10 classes with the highest relative abundance in the two groups.

At the phylum level, the composition of cecal bacterial communities in the two laying groups was dominated by three major phyla: *Bacteroidota* (51.00% vs. 58.00%), *Firmicutes* (25.20% vs. 20.90%), and *Desulfobacterota* (5.40% vs. 6.20%), collectively accounting for over 82% of the total bacterial abundance in both groups (Figure 4A,B). Venn diagram analysis revealed that at the class level, groups a and b shared 27 bacterial classes, with 12 classes showing significant differences between the two groups (Figure 4C). Further analysis of the top 10 most abundant bacterial classes is presented in Figure 4D. The relative abundances of *Bacteroidia*, *Methanobacteria*, *Fusobacteriia*, *Negativicutes* and *Bacilli* were higher in Group b than in Group a, while the relative abundances of *Clostridia*, *Desulfovibrionia*, *Spirochaetia*, *Coriobacteriia*, and *Deferribacteres* were higher in Group a than in Group b. At the genus level, the top 10 bacterial taxa were clustered in each group (Figure 5A,B). The relative abundances of *Bacteroides*, *Methanobrevibacter*, *Butyricimonas* and *Fusobacterium* were higher in Group b than in Group a, while the relative abundances of *Desulfovibrio*, *Treponema*, *Spirochaetia*, *Prevotella_9*, *Olsenella*, *Prevotellaceae_ucg-001* and *Prevotella_7* were higher in Group a than in Group b.

### 3.4. Functional Predictions Based on the Gut Microbiota of Caged Laying Ducks

**Figure 6:**
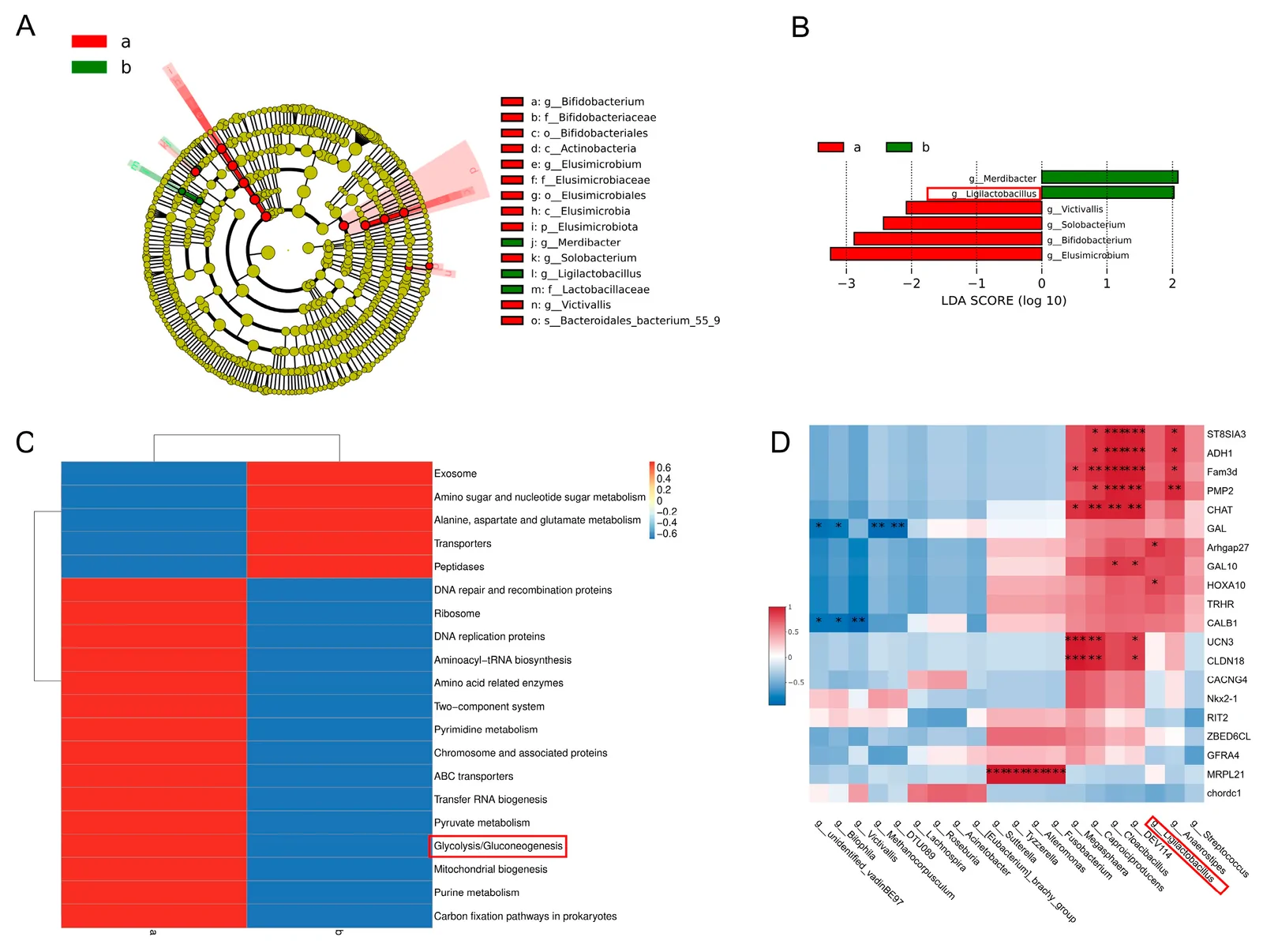
(**A**) Cladogram of the main taxa of microbiota that were different between high− and low−laying groups. (**B**) LEfSe analysis (taxa with LDA score ≥ 2, *p* < 0.05); color (red to green) indicates the correlation (positive to negative). (**C**) Top10 KEGG heatmap based on Tax4Fun functional analysis. (**D**) Correlation analysis between the microbiota and key genes. * represents the correlation strength was significant (*p* < 0.05), ** represents the correlation strength was extremely significant (*p* < 0.01), and *** represents the correlation strength was extremely significant (*p* < 0.001).

To identify cecal microbial biomarkers with both statistical and biological relevance between high- and low-laying Muscovy duck groups, microbial contributions between the two groups were evaluated using the LDA score. The evolutionary branches of biomarkers at all levels are shown in Figure 6A. The results of the cecal comparison are presented in Figure 6B. At the genus level, the relative abundances of *Ligilactobacillus* and *Merdibacter* were significantly higher in Group b; whereas in Group a, the relative abundances of *Elusimicrobium*, *Bifidobacterium*, *Victivallis*, *unidentified_vadinBE97*, and *CHKCI002* were significantly increased (*p* < 0.05). Tax4Fun-based functional prediction identified the top 20 KEGG pathways, as shown in Figure 6C. Upon comparison with the top 20 KEGG pathways derived from transcriptomic analysis, only one pathway, glycolysis/gluconeogenesis, was found to be commonly enriched between the two omics datasets, making it a candidate pathway for further integrative investigation. Spearman’s correlation analysis (Figure 6D) revealed positive correlations between the top 20 genera and top 20 DEGs. Among these, seven genera exhibited significantly positive correlations with 11 genes, while 6 genera showed significantly negative correlations with two genes (*p* < 0.05). Additionally, we observed that *Ligilactobacillus*, with significant differences between the two groups, was significantly positively correlated with *Arhgap27* and *HOXA10*.

## 4. Discussion

The orchestration of egg production in poultry involves a complex array of physiological inputs. Mounting evidence indicates a bidirectional interplay between the intestinal microbiome and the endocrine system, which synergistically modulates reproductive phenotypes. However, previous investigations have largely concentrated on successional shifts in microbial community structure during the laying or broodiness period, leaving the regulatory crosstalk with the host ovarian transcriptome largely uncharted. To delineate these intricate networks, we performed parallel microbiome and transcriptomic profiling on cecal contents and ovarian tissues, comparing Muscovy ducks with divergent laying performances. Our phenotypic characterization revealed that the high-reproduction line (Group a) exhibited a significantly superior egg-laying performance compared to the ordinary population (Group b) (24.60 ± 9.22 vs. 15.87 ± 6.64 eggs, *p* < 0.01). Moreover, ovarian morphological analysis confirmed that Group a possessed larger ovaries and a more abundant follicular hierarchy, with significantly higher follicle counts at all developmental stages (*p* < 0.01). RNA sequencing data analysis was performed to compare the ovaries of the high- and low-laying Muscovy ducks, revealing 823 DEGs (363 upregulated and 460 downregulated). To preliminarily uncover the biological functions of the identified DEGs, we performed KEGG enrichment analysis and curated the top 20 pathways based on their statistical significance. This analysis yielded several canonical signaling pathways known to govern ovarian physiology, notably the MAPK, Calcium, Notch, and Wnt signaling pathways, consistent with a previous transcriptomic study on white Muscovy ducks [24], reinforcing their fundamental roles in avian ovarian function. Furthermore, based on this transcriptomic profiling, we further performed an integrative correlation analysis between the cecal microbial communities and the DEGs to identify signaling pathways that were commonly enriched in both omics datasets. This joint analysis highlighted the glycolysis/gluconeogenesis pathway as a convergently enriched signaling cascade shared between the gut microbiome and the ovarian transcriptome. The biological relevance of this pathway to reproductive function is well substantiated by previous studies. In poultry, glycolysis has been established as the metabolic foundation for follicular development and the maintenance of normal ovarian function. Specifically, granulosa cell glycolysis has been identified as the primary energy source driving follicular growth [25]. Yang et al. [26] reported that the glycolysis/gluconeogenesis pathway may be linked to fertility phenotypes in geese, further reinforcing the potential role of glycolytic metabolism in avian reproductive performance. This glycolytic dependency appears to be conserved across vertebrates, as evidenced by mammalian studies showing that primordial follicle activation is accompanied by upregulated expression of glycolysis-related genes, suggesting enhanced glycolytic activity during this process [27]. Collectively, these findings lend credence to the possibility that the glycolysis/gluconeogenesis pathway, identified through our multi-omics integration, may contribute to the regulation of ovarian function and, consequently, laying performance in ducks.

16S rRNA gene sequencing analysis revealed significant alterations in the richness and diversity of cecal microbial communities between duck groups with divergent laying performance. While *Bacteroidota* and *Firmicutes* dominated the cecal microbiota in both Group a and Group b, their relative abundance ratios differed significantly between the two groups. Specifically, the relative abundances of *Bacteroidota* were 51.00% and 58.00%, and those of *Firmicutes* were 25.20% and 20.90% in Group a and Group b, respectively, yielding an elevated *Firmicutes*/*Bacteroidota* (F/B) ratio (49.41% vs. 36.03%). This compositional shift is consistent with the hypothesis that high-yielding birds experience low-grade metabolic inflammation during peak laying. Mechanistically, the F/B ratio is recognized as a proxy for energy-harvesting capacity [28], yet in the context of high egg production, its elevation likely reflects a maladaptive shift toward pro-inflammation. This inference is supported by recent evidence demonstrating that stressed poultry consistently exhibit a marked increase in the F/B ratio [29,30,31]. In our high-yield group, the chronic metabolic burden associated with intensive egg production, including hepatic oxidative stress and increased feed intake, may compromise intestinal barrier integrity, thereby establishing a self-perpetuating low-grade pro-inflammatory milieu. Collectively, these findings suggest that the elevated F/B ratio in high-yielding Muscovy ducks may reflect a potential association between gut microbiota composition and laying performance. Furthermore, integrative analysis of the transcriptomic and gut microbial datasets screened out *Ligilactobacillus* as a potential key gut microorganism associated with laying performance traits in Muscovy ducks. This bacterial genus has been reported to participate in estrogen degradation and conjugation [32,33], thereby potentially influencing circulating estrogen levels, which are closely correlated with laying performance. Among its members, *Ligilactobacillus salivarius* [34] has been shown to enhance egg production in laying hens via modulation of intestinal microbiota and lipid metabolism [35,36]. However, in the present study, the cecal abundance of *Ligilactobacillus* was significantly higher (*p* < 0.05) in the low-laying group than in the high-laying group of Muscovy ducks, implying that *Ligilactobacillus* may exert different functions across different host species or physiological states. Interestingly, both the elevated F/B ratio and the altered *Ligilactobacillus* abundance may be linked to the glycolysis/gluconeogenesis pathway identified in our multi-omics integration. The increased F/B ratio may provide glycolytic substrates (e.g., propionate and acetate), while the lower *Ligilactobacillus* abundance in high-laying ducks may affect ovarian glycolysis via endocrine-metabolic crosstalk. Given that glycolysis fuels avian ovarian function [24,25], these microbial features may ultimately influence laying performance through this pathway, warranting further investigation.

Based on Spearman’s correlation analysis, 11 DEGs were identified as significantly correlated with microbial genera that differed between the two groups (*p* < 0.05). Among these, *Arhgap27* and *HOXA10* attracted our particular interest due to their significant correlations with *Ligilactobacillus* abundance. As a member of the Rho GTPase-activating protein family, *Arhgap27* is involved in the control of small GTPase signaling and has been linked to clathrin-mediated endocytic pathways [37]. This functional versatility raises the possibility that *Arhgap27* may also contribute to reproductive physiology. Mathieson et al. [38] performed a GWAS on 785,604 European individuals and identified 43 loci associated with reproductive success, with *Arhgap27* missense variants linked to greater offspring number but earlier reproductive decline, indicating a trade-off between fertility and reproductive aging. *HOXA10* (Homeobox A10) [39] encodes a sequence-specific DNA-binding transcription factor whose expression is finely regulated by sex steroids such as estradiol and progesterone, underscoring its potential role in hormone-responsive reproductive tissues. In humans, the expression of *HOXA10* is restricted to primordial and early primary follicles, implying a potential role in the initiation of follicular development [40]. In poultry, transcriptomic profiling of ovarian tissues from Muscovy ducks at early-, peak-, and late-laying stages revealed that *HOXA10* was differentially expressed across these phases, suggesting its involvement in ovarian maturation and laying performance [41]. This observation is further corroborated by a recent study in chickens, where *HOXA10* was identified as a core transcription factor within the regulatory network governing follicular development across six progressive stages, from small white follicles to pre-ovulatory follicles [42]. In alignment with these findings, our study detected a significant correlation between *ARHGAP27* and *HOXA10* expression and *Ligilactobacillus* abundance in duck cecal samples, raising the possibility that these genes could bridge gut microbial ecology and host ovarian function. However, the underlying regulatory mechanisms through which these two genes and gut microbiota collectively modulate laying performance in Muscovy ducks remain largely elusive. Future studies employing integrative multi-omics approaches, functional validation in vivo, and targeted microbial intervention experiments are warranted to elucidate the causal relationships and molecular cascades linking gut microbial ecology, host gene expression, and reproductive physiology.

## 5. Conclusions

In summary, by integrating ovarian transcriptomic and cecal microbial profiling of Muscovy ducks with divergent laying performance, we correlated *Ligilactobacillus* abundance with reproduction-related genes (*Arhgap27* and *HOXA10*) and identified the glycolysis/gluconeogenesis pathway as a potential gut–ovary metabolic link. These findings provide novel insights into host–microbe interactions underlying reproductive efficiency.

## Data Availability

The original data presented in this study are openly available in the Genome Sequence Archive (GSA) at the National Genomics Data Center (https://ngdc.cncb.ac.cn), under accession number PRJCA044169.

## Supplementary Materials

The following supporting information can be downloaded at: https://www.mdpi.com/article/10.3390/vetsci13080816/s1, Figure S1: Taxonomic annotation stacked bar chart; Table S1: The composition and nutrient levels of the basal diets (air-dry basis); Table S2: Primer information for qRT-PCR; Table S3: Quality evaluation and analysis of preprocessing RNA-Seq data; Table S4: Number of reads that passed through each step of paired-end reads assembly and quality control.
