# Supplementary material for: Integrated Multi-Omics Reveals Gut Microbiota-Ovarian Crosstalk of Laying Performance Between High- and Low-Laying Muscovy Ducks
Source: Vet Sci. 2026 Aug 17;13(8):816. doi: 10.3390/vetsci13080816 (PMC13517328; doi:10.3390/vetsci13080816)
Supplement: Supplementary file 1 [file vetsci-13-00816-s001.zip › Table S1 The composition and nutrient levels of the basal diets (air-dry basis).pdf]

Table S1 The composition and nutrient levels of the basal diets (air-dry basis)

Diet Composition		Nutrient level	
Items	Composition%	Items	Content
Corn	54.50	Metabolizable energy MJ/kg	12.35
Soybean	23.80	Crude protein %	17.85
Wheat Bran	9.20	Curde fiber %	3.84
Limestone (Calcium Carbonate)	8.00	Lysine %	0.74
Soybean oil	1.20	Methionine %	0.42
Calcium Hydrogen Phosphate	0.86	Ca %	3.08
Salt	0.04	P %	0.68
Premix	2.40	Sodium %	0.18

Note: The premix provides the following per kilogram of diet: iron 70 mg, copper 12 mg, zinc 80 mg, manganese 120 mg, iodine 1.0 mg, vitamin A 12,000 IU, vitamin D_3 3,200 IU, vitamin E 300 IU, vitamin K_3 40 mg, vitamin B_1 3.0 mg, vitamin B_2 5.0 mg, vitamin B_6 3.5 mg, vitamin B_{12} 0.04 mg, nicotinic acid 35 mg, folic acid 1.5 mg, biotin 0.8 mg.
