# Supplementary material for: Integrated Multi-Omics Reveals Gut Microbiota-Ovarian Crosstalk of Laying Performance Between High- and Low-Laying Muscovy Ducks
Source: Vet Sci. 2026 Aug 17;13(8):816. doi: 10.3390/vetsci13080816 (PMC13517328; doi:10.3390/vetsci13080816)
Supplement: Supplementary file 1 [file vetsci-13-00816-s001.zip › Table S2 Primer information for qRT-PCR.pdf]

Table S2 Primer information for qRT-PCR

Gene	Sequence (5′-3′)	Tm (°C)	Product length (bp)
PMP2	F: AAAGAACTAGGAGTGGGCTT<br>R: GTCATCTGCTGTCGTTTCAT	59	171
CHAT	F: CACGCTCTCGCCTGACCTTG<br>R: GTCCTTATCAATCCCAATCGCTTCTG	59	152
GAL	F: CACTACCTCCTGACGCTGTTCTC<br>R: CGCTCCTCGTAGATGACCATGATC	59	133
CALB1	F: TGTGGACCAATATGGCAAAG<br>R: AATTCTCCTCCGTCGGTAAT	59	80
β-ACTIN	F：GGAAGTTACTCGCCTCTC<br>R：CGCTCGCTGAACAAATC	60	155
GADPH	F: ATCTTTCTTGGGTATGGAGTC<br>R:TTTCATCGTGCTGGGTG	58	185
