# Supplementary material for: Integrated Multi-Omics Reveals Gut Microbiota-Ovarian Crosstalk of Laying Performance Between High- and Low-Laying Muscovy Ducks
Source: Vet Sci. 2026 Aug 17;13(8):816. doi: 10.3390/vetsci13080816 (PMC13517328; doi:10.3390/vetsci13080816)
Supplement: Supplementary file 1 [file vetsci-13-00816-s001.zip › Table S3 Quality evaluation and analysis of preprocessing RNA-Seq data.pdf]

Table S3 Quality evaluation and analysis of pre-processing RNA-Seq data

sample	raw_reads	raw_bases	clean_reads	clean_bases	Q30	GC_pct
a1	46540362	6.98G	44230232	6.63G	93.61	49.89
a2	44480674	6.67G	42577352	6.39G	93.42	49.54
a3	47363748	7.1G	43732408	6.56G	93.65	50.03
b1	46253908	6.94G	41633694	6.25G	94.08	51.03
b2	45936860	6.89G	43453570	6.52G	93.33	47.10
b3	43482166	6.52G	42813240	6.42G	92.81	47.11
