# Supplementary material for: Integrated Multi-Omics Reveals Gut Microbiota-Ovarian Crosstalk of Laying Performance Between High- and Low-Laying Muscovy Ducks
Source: Vet Sci. 2026 Aug 17;13(8):816. doi: 10.3390/vetsci13080816 (PMC13517328; doi:10.3390/vetsci13080816)
Supplement: Supplementary file 1 [file vetsci-13-00816-s001.zip › Table S4 Number of reads that passed through each step of paired-end reads assembly and quality control.pdf]

Table S4 Number of reads that passed through each step of paired-end reads assembly and quality control

Sample	RawPE	Combined	Qualified	Nochime	Base(nt)	Avglen(nt)	GC	Q20	Q30	ASV
a1	81522	81387	81024	74879	18936818	252.90	53.94%	99.26%	97.34%	605
a2	59673	59557	59275	54648	13818686	252.87	53.31%	99.18%	97.09%	553
a3	83284	83148	82759	75440	19080260	252.92	53.67%	99.25%	97.28%	719
b1	60315	60151	59907	56883	14487380	254.69	51.10%	99.24%	97.31%	235
b2	140142	139044	138611	125593	31764847	252.92	53.08%	99.42%	97.86%	1067
b3	64854	64736	64472	60596	15331968	253.02	52.08%	99.21%	97.21%	583
